# Oral Pulse Betamethasone, Methotrexate, and Combination Therapy to Treat Severe Alopecia Areata: A Randomized, Double-blind, Placebo-controlled, Clinical Trial

**DOI:** 10.22037/ijpr.2020.113868.14536

**Published:** 2021

**Authors:** Ali Asilian, Farahnaz Fatemi, Zakiye Ganjei, Amir Hossein Siadat, Fatemeh Mohaghegh, Mansour Siavash

**Affiliations:** a *Skin Diseases and Leishmaniasis Research Center, Department of Dermatology, Isfahan University of Medical Sciences, Isfahan, Iran. *; b *Endocrine and Metabolism Research Center, Isfahan University of Medical Sciences, Isfahan, Iran.*

**Keywords:** Alopecia areata, Methotrexate, Corticosteroid, Randomized controlled trial

## Abstract

The purpose of this study is to compare oral betamethasone pulse therapy, methotrexate therapy and a combination of the two for patients with Alopecia Areata (AA) as an autoimmune disorder. In this study, 36 patients with severe AA were selected and classified into three groups of 12: 1- Oral betamethasone therapy (3 mg, once a week) with placebo; 2- Oral methotrexate (15 mg, once a week) with placebo; and 3- A combination of methotrexate (15 mg, once a week) and betamethasone (3 mg, once a week). The Severity Alopecia Tool (SALT) was used to measure improvements in the lesions through photographs, and the patients also rated their condition on the Visual Analogue Scale (VAS). Assessments were performed, and the results were compared at baseline and then at intervals of three months for nine months. The demographics and SALT score were similar in the three groups (*P > *0.05). All the groups showed improvements in SALT, VAS and photographic scores three months after beginning the treatment (*P < *0.001). Betamethasone therapy (*P = *0.006) and combination therapy (*P < *0.001) provided greater SALT improvement than methotrexate, and combination therapy led to a greater improvement in VAS and photographic findings compared to the two other groups (*P < *0.05). Oral steroid, methotrexate and combination pulse therapy were effective treatments for AA, while oral steroid pulse therapy and combination therapy were superior to methotrexate.

## Introduction

Alopecia Areata (AA) is an autoimmune disorder and cosmetically disfiguring condition observed in about 1% of outpatient dermatology visits(1) and about 0.1% to 0.2% of the global population (2). Different treatment methods are used for this disorder (1, 3). AA patients may be treated with local corticosteroid injections or systemic steroids, or immunomodulators such as cyclosporine or azathioprine. Some patients experience recurrent courses or a refractory form of the disease (4-6). Methotrexate, in doses of 7.5 to 20 mg, has been used for a wide variety of inflammatory skin diseases (7-9). Limited studies have reported that methotrexate is used to treat alopecia. Systemic steroids can also be effective for the treatment of AA (1, 10). This study assessed the efficacy of combination treatment with methotrexate and betamethasone versus corticosteroids or methotrexate alone.

## Experimental

*Study design*

This randomized, double-blind, placebo-controlled, parallel-group trial was conducted on 36 patients with severe AA at Al-Zahra Hospital and Sedigheh Tahereh Research Center, affiliated to Isfahan University of Medical Sciences in Isfahan, Iran. The study was registered at the Iranian Registry of Clinical Trials with the code IRCT20181226042136N1 and approved by the Ethics Committee of Isfahan University of Medical Sciences with the reference number IR.MUI.MED.REC.1397.146.

*Patients*

The patients with the following criteria were included in the study: 1- Over 50% of scalp involvement; 2- Age range of 16-60 years; 3- Refractory disease with local and systemic therapies, which was defined as unresponsiveness to systemic therapies for over six months; and 4-Patients’ consent to participate in the study. The patients with the following criteria were excluded from the study: 1- Pregnancy or lactation; 2- Hemoglobin level less than 9 mg/dL; 3- White blood cell count under 4000 mm^3^; 4-Platelet level under 100000 mm^3^; 5- Blood potassium level less than 3.5 mEq/L; 6-Over two-fold increase in hepatic enzyme levels more than the normal limit; 7- Increased Erythrocyte Sedimentation Rate (ESR); 8- A positive stool exam for *Strongyloides stercoralis*; 9- Positive tests for viral hepatitis; and 10- The presence of contraindications for oral betamethasone use (including diabetes, hypertension, hypersensitivity to drugs, presence of active infection, a recent history of bone surgery, peptic ulcer, psychosis or depression) and hypersensitivity to methotrexate. The sample size was calculated as 12 per group.

*Randomization*

The randomization sequence was created by block randomization using the Random Allocation Software. The patients were randomly classified into three groups at a ratio of 1:1:1, including (A) The betamethasone therapy group, (B) The methotrexate therapy group and (C) The combination therapy with betamethasone plus methotrexate group. 

*Interventions*

Group A was treated with oral betamethasone pulse therapy as 0.5-mg pills (Iran Hormone Company, Iran) in doses of 1 mg, three times a day, one day a week on Saturdays, with a matching placebo of methotrexate taken on Fridays. This treatment was performed once a week for six months (11). Group B was treated with a weekly 15 mg of methotrexate as 2.5-mg pills (Ebetrex, EBEWE Company, Austria), in doses of 5 mg, three times a day, one day a week on Fridays, with a matching placebo of betamethasone taken on Saturdays, for six months.

**Figure 1:**
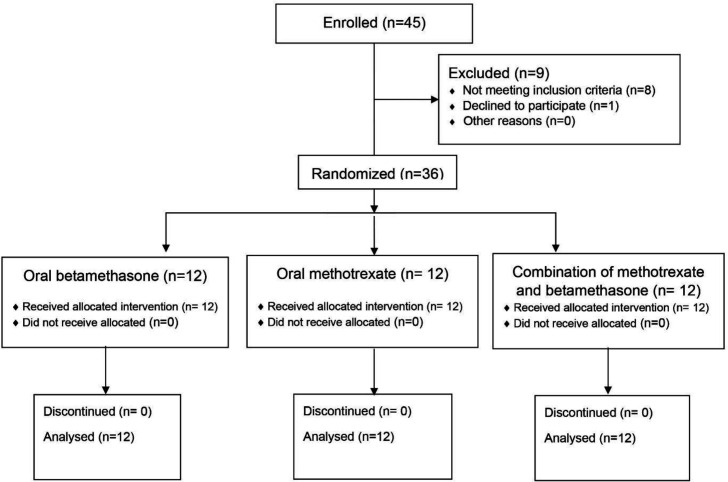
Patient disposition (CONSORT flow chart).

Group C was treated by combining the methotrexate and betamethasone therapies. They received 15 mg of methotrexate, divided into three doses of 5 mg on Fridays, and 3 mg of betamethasone, divided into three doses of 1 mg, on Saturdays, for a duration of six months (Figure 1). 

In all the groups, 1 mg of folic acid was prescribed daily, except for the days of methotrexate consumption. All the patients and investigators were blinded to the treatment groups.

*Assessments*

Demographic information (age and gender), duration of AA, previously-received treatments, history of osteoporosis, psychiatric disorder history and laboratory findings, including complete blood count, liver enzyme levels, renal function tests, viral hepatitis tests, serum potassium levels, ESR, fasting blood sugar and stool exam, were recorded at baseline. Disease severity was assessed at baseline and then every three months for nine months, from January 2017 to March 2018. 

The severity of disease was scored using three methods: 

1- The Olsen/Canfield tool or the Severity Alopecia Tool (SALT) was operated, and the SALT score was calculated by measuring the percentage of hair loss in each of the four areas of the scalp —vertex 40%, right profile 18%, left profile 18%, and posterior aspect 24%— and adding the total to achieve a composite score. Hair regrowth was defined by a decrease in the SALT score (12). The amount of involvement of each of the aforementioned areas was measured, and the percentage of areas was summed up to measure the total area of alopecia. 

2- Photographic scoring system: Before the treatment, standard photographs(13) were taken of all the patients using a Digital SONY Camera, 16 Megapixel, Japan. The photos were captured in four views: The vertex, mid-pattern, temporal and frontal views. Three dermatologists who were blinded to the group allocations used a 7-point scale to grade the patients’ photographs (14):

Highly decreased = -3

Moderately decreased = -2

Slightly decreased = -1

No change = 0

Slightly increased = +1

Moderately increased = +2

Highly increased = +3

3- The patients were also requested to grade their lesions’ improvement on a Visual Analogue Scale (VAS) (15). 

*Statistical analysis*

SPSS software (SPSS version 20, IBM, United States) and statistical tests including the Chi-square test, ANOVA, Fisher’s exact test, Friedman’s test, Kruskal-Wallis test and Generalized Estimating Equation (GEE) were used to analyze the collected data. *P*-values less than 0.05 were considered statistically significant.

## Results

There were no significant differences among the three groups in terms of age, gender, and marital status, duration of being affected by the disease, presence of concurrent skin diseases, medical history and previously-received treatments (Table 1, *P > *0.05).

**Figure 2:**
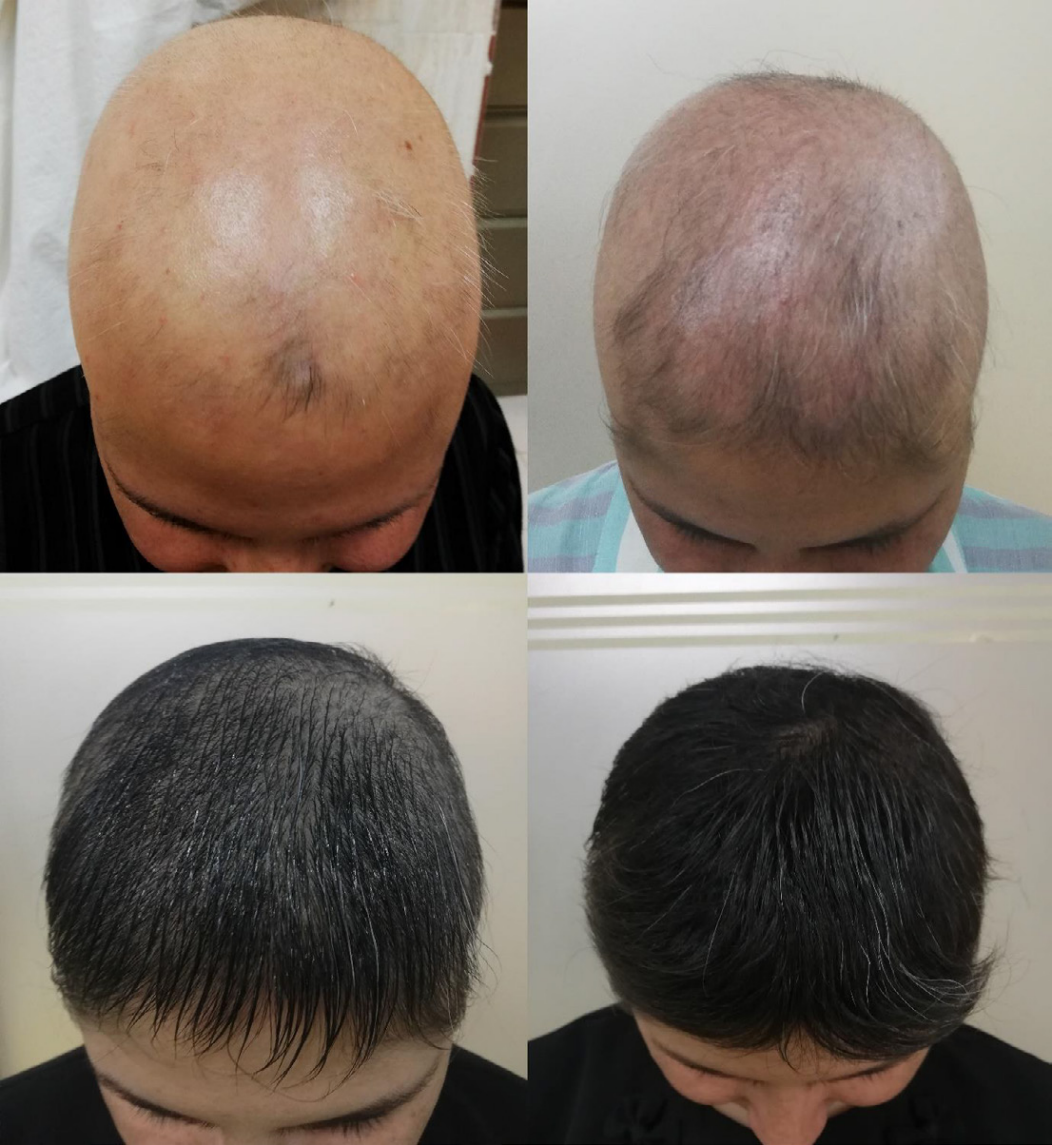
Disease improvement in the combination therapy group after nine months of treatment. (A) Pretreatment, (B) Three months after the treatment, (C) Six months after the treatment, and (D) Nine months after the treatment

All the groups showed improvements in SALT, VAS and photographic scores three, six and nine months after beginning the treatment, respectively (Table 2), especially the combination therapy group (Figure 2). Betamethasone therapy (*P = *0.006) and combination therapy (*P < *0.001) provided greater SALT improvement than methotrexate within nine months, as the amount of reduction was 43% in the SALT score in the combination therapy group, 26% in the betamethasone group and 23% in the methotrexate group, and there was no significant difference between the betamethasone group and the combination therapy group in this respect (*P > *0.05). Combination therapy led to greater improvements in the VAS score within nine months compared to betamethasone (*P = *0.015) and methotrexate (*P < *0.001), as there were six points of increase in the combination therapy group, four in the methotrexate group and three in the betamethasone group. There were significant differences among the groups in terms of photographic scores within the time, as there were two points of increase in the photographic score in the combination therapy group and one point in the other two groups, and the photographic findings of the combination therapy were superior to betamethasone (*P = *0.019) and methotrexate (*P < *0.001) treatments alone. 

During the study, no patients showed any serious complications resulting in the stoppage of the treatment or exclusion from the study. Only one patient in the methotrexate group and another patient in the group that received betamethasone and methotrexate showed gastrointestinal symptoms, which were all rehabilitated by the daily consumption of folic acid.

**Table 1:** Comparison of demographic and alopecia features among three groups

**Variable**	**Betamethasone**	**Methotrexate**	**Betamethasone+Methotrexate**	***P*** **-value**
**Gender**				
Female	5 (41.7%)	8 (66.7%)	8 (66.7%)	0.35
Male	7 (58.3%)	4 (33.3%)	4 (33.3%)	
Age	27.50 ± 9.30	31.25 ± 9.30	25.83 ± 8.15	0.32
Duration of disease	4.50 ± 2.97	6 ± 4.63	6.42 ± 5.55	0.55
**Marital status**				
Single	8 (66.7%)	6 (50%)	8 (66.7%)	0.75
Married	4 (33.3%)	6 (50%)	4 (33.3%)	
**Presence of concurrent skin disease**				
Negative	11 (91.7%)	9 (75%)	9 (75%)	0.76
Atopic dermatitis	1 (8.3%)	2 (16.7%)	1 (8.3%)	
Vitiligo	0 (0%)	1 (8.3%)	2 (16.7%)	
**Previous medical history**				
Positive	2 (16.7%)	3 (25%)	4 (33.3%)	0.88
Negative	10 (83.3%)	9 (75%)	8 (66.7%)	
**Previous treatments**				
Local cortone	12 (100%)	12 (100%)	11 (91.7%)	0.99
Systemic cortone	12 (100%)	12 (100%)	11 (91.7%)	0.99
Intralesion cortone	0 (0%)	0 (0%)	0 (0%)	-
Methotrexate	3 (25%)	1 (8.3%)	1 (8.3%)	0.58
Azathioprine	6 (50%)	7 (58.3%)	5 (41.7%)	0.71
Phototherapy	4 (33.3%)	2 (16.7%)	0 (0%)	0.13
Cyclosporine	1 (8.3%)	6 (50%)	5 (41.7%)	0.08
Renunciaron	4 (33.3%)	7 (58.3%)	10 (83.3%)	0.05

**Table 2:** Comparison of SALT score, photographic scores and visual analog scale findings among three groups within the time

**Treatment group**	**Prior to treatment**	**Within 3 months**	**Within 6 months**	**Within 9 months**	***P*** **-value** **(within groups)**
**Disease SALT score** ^a^					
Beamethasone	100% (100-100)	79.50% (68.83-89.25)	63% (40.75-75.16)	74% (64.16-88.41)	<0.001
Methotrexate	100% (100-100)	90.83% (81.16-100)	78% (68.33-100)	77.66% (72-100)	<0.001
Betamethasone+Methotrexate	100% (100-100)	79.50% (63.83-89)	61.83% (24.41-61.83)	57% (37.33-77.66)	<0.001
*P*-value (between groups)	-	0.044	0.059	0.078	
**Photographic scores** ^b^					
Beamethasone	0 (0-0)	1 (0.5-1)	1 (1-2)	1 (0.5-1.25)	<0.001
Methotrexate	0 (0-0)	0.5 (0-1)	1 (0-1)	1 (0-1)	<0.001
Betamethasone+Methotrexate	0 (0-0)	1 (0.5-1)	2 (1-2.5)	2 (1-2)	<0.001
*P*-value (between groups)	-	0.11	0.073	0.12	
**Visual analog scale (0-10)**					
Beamethasone	0 (0-0)	3 (3-4)	5 (4.25-7)	3 (1.25-4)	<0.001
Methotrexate	0 (0-0)	2.5 (0-3)	4 (0-5)	4 (0-5)	<0.001
Betamethasone+Methotrexate	0 (0-0)	3.75 (0.75-5)	6 (3.25-8)	6 (4.25-7)	<0.001
*P*-value (between groups)	-	0.15	0.14	0.007	

^a^Decrease in SALT score reflects improvement in the disease.

^b^Increase in photographic score reflects improvement in the disease.

## Discussion

AA negatively affects the emotional and social life of patients. About 60% of patients experience their first episode of AA before their 20’s (10). Some patients have the refractory form of the disease that worsens the disease status and is challenging for dermatologists to manage (16). This randomized, double-blind, placebo-controlled trial was designed to compare betamethasone and methotrexate and their combination in the treatment of AA. 

All the three assessed groups showed significant improvements, but steroid pulse therapy and combination therapy were more effective than methotrexate therapy alone. These findings are consistent with previous studies, in which AA improved with steroid pulse therapy (1, 17-20). methotrexate pulse therapy (21) and their combination (22, 23). 

MTX is an effective treatment for severe AA and regrown hair when used in combination with low to moderate doses of corticosteroids (24). While hair may regrow with combination therapy, adverse effects related to the long-term use of methotrexate should be carefully considered (23). The present study results are consistent with previous reports about the better responsiveness of patients to steroids compared to methotrexate (21). Some studies have revealed that the significant therapeutic effects of methotrexate in combination with steroids require long-term treatment (22, 25). Therefore, a hypothesis has been raised that the better efficacy of methotrexate therapy can be achieved by long-term treatment. 

The other regimen, which included only methotrexate, was associated with acceptable outcomes with a similar required duration of 2.5 to 3 months for hair regrowth. All the studies on the subject had shown that doses less than 20 mg per week were adequate for this good response, and they nearly unanimously reported the good tolerance of this dose with minimum adverse effects (21, 24 and 26).

## Conclusion

Oral pulse steroid, methotrexate, and combination therapy were all effective for AA; however, steroid pulse therapy and combination therapy were more effective than methotrexate alone. The better efficacy of methotrexate therapy can be achieved by long-term treatment; more studies are therefore recommended to be conducted with longer durations of follow-up. 

## Study limitations

Some of the patients’ expectations were fully recovered; also, the number of patients with severe alopecia areata who met the inclusion criteria was limited. Another limitation was the patients’ concerns about the side-effects of their medications.

## Funding

This study was funded by Isfahan University of Medical Sciences.
